# Effects of Intervention Timing on Health-Related Fake News: Simulation Study

**DOI:** 10.2196/48284

**Published:** 2024-08-07

**Authors:** Nahyun Gwon, Wonjeong Jeong, Jee Hyun Kim, Kyoung Hee Oh, Jae Kwan Jun

**Affiliations:** 1 Cancer Knowledge and Information Center National Cancer Control Institute National Cancer Center Goyang Republic of Korea

**Keywords:** disinformation, fenbendazole, cancer information, simulation, fake news, online social networking, misinformation, lung cancer

## Abstract

**Background:**

Fake health-related news has spread rapidly through the internet, causing harm to individuals and society. Despite interventions, a fenbendazole scandal recently spread among patients with lung cancer in South Korea. It is crucial to intervene appropriately to prevent the spread of fake news.

**Objective:**

This study investigated the appropriate timing of interventions to minimize the side effects of fake news.

**Methods:**

A simulation was conducted using the susceptible-infected-recovered (SIR) model, which is a representative model of the virus spread mechanism. We applied this model to the fake news spread mechanism. The parameters were set similarly to those in the digital environment, where the fenbendazole scandal occurred. NetLogo, an agent-based model, was used as the analytical tool.

**Results:**

Fake news lasted 278 days in the absence of interventions. As a result of adjusting and analyzing the timing of the intervention in response to the fenbendazole scandal, we found that faster intervention leads to a shorter duration of fake news (intervention at 54 days = fake news that lasted for 210 days; intervention at 16 days = fake news that lasted for 187 days; and intervention at 10 days = fake news that lasted for 157 days). However, no significant differences were observed when the intervention was performed within 10 days.

**Conclusions:**

Interventions implemented within 10 days were effective in reducing the duration of the spread of fake news. Our findings suggest that timely intervention is critical for preventing the spread of fake news in the digital environment. Additionally, a monitoring system that can detect fake news should be developed for a rapid response

## Introduction

The development of Social Network Services (SNS) has enabled people to gather information anytime and anywhere through various channels, while also allowing anyone to post and disseminate content easily. However, this has led to increased consumption of unclear information and the spread of disinformation, causing widespread confusion [1]. Social media has become a powerful source for fake news dissemination, significantly impacting society as manipulated and false content is easier to generate and harder to detect, with disinformation actors continually changing their tactics [2]. This issue extends to health-related information as well [3]. For instance, the spread of COVID-19, the global pandemic of 2020, was greatly influenced by fake news propagated via SNS [4,5]. As an example, holding your breath for 10 seconds to 1 minute is not a COVID-19 self-test and can be dangerous [2].

**Figure 1:**
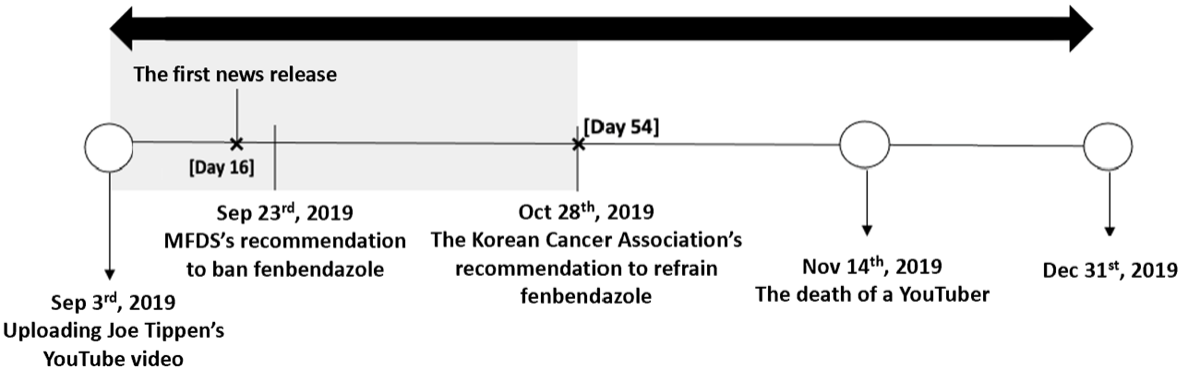
Timeline of Key issues throughout 2019 regarding fenbendazole. From the day the fake news was first uploaded, the response dates of the relevant major agencies were identified as key issues. MFDS: Ministry of Food and Drug Safety.

**Figure 2:**
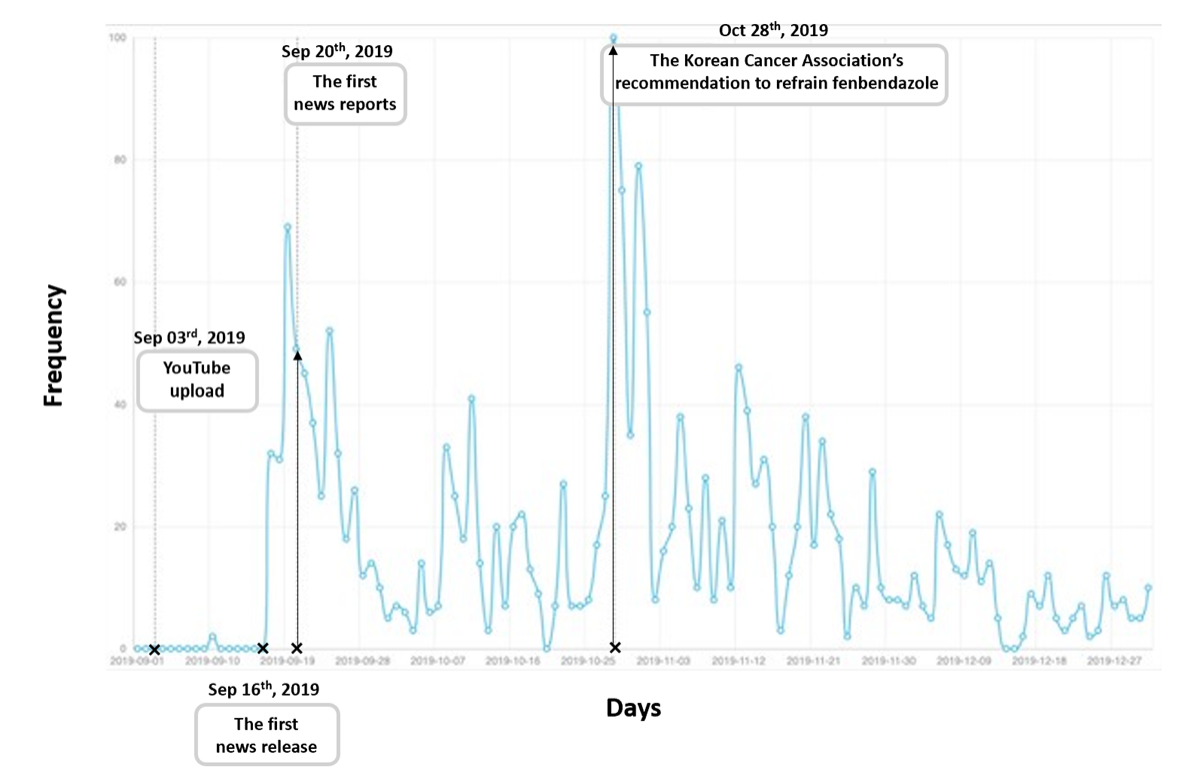
The results of searching for “fenbendazole” on Google Trends in Korea. The x-axis represents the period from September 2019, when fenbendazole-related issues were first mentioned, to December 2019. The y-axis indicates the search frequency, with the highest frequency point normalized to 100.

One notable case of damage caused by cancer-related health fake news in South Korea involved fenbendazole, a dog anthelmintic (Figure 1). On September 3, 2019, a YouTube video claimed that a man named Joe Tippens cured his cancer by taking fenbendazole. This video rapidly spread among patients with cancer, leading to a surge in demand for fenbendazole and its high-priced illegal distribution nationwide. Twenty days after the initial report, the Ministry of Food and Drug Safety (MFDS), the national agency responsible for ensuring the safety of food and drugs in South Korea, banned the use of fenbendazole, warning of its serious potential harm [6]. On October 28, which was 54 days after the first report, the Korean Cancer Association, the most prestigious cancer-related association in South Korea, released its first official statement on the incident, marking its initial public stance on this health issue (Figure 2).

Despite warnings from medical experts, the controversy continued among fake news believers for over a year. The issue gradually diminished following continuous alerts from experts and the death of the YouTuber who had initially spread the misinformation, as he passed away without being fully cured. This case highlights the severity of the fake news problem.

Researchers studying fake news urge related organizations to intervene to prevent their spread [7-9]. However, despite the intervention of related organizations, such as the MFDS and the Korean Cancer Association, the fenbendazole scandal continued. Therefore, this study examines the appropriate timing for interventions by relevant organizations in the case of fake news. As barriers to content creation and consumption decrease with the spread of SNS, the damage caused by fake news is expected to increase [10]. Given the unpredictable spread of fake news, witnessed in events like the fenbendazole case, effective interventions by relevant organizations become paramount. However, research into the optimal timing of such interventions to mitigate the transmission of fake news remains insufficient. Hence, this study aims to elucidate the imperative of immediate interventions by pertinent organizations in curtailing the dissemination of false information and pinpointing opportune moments for such actions. To this end, the study endeavors to simulate the spread of fake news on an actual SNS within the context of the fenbendazole incident and assess the timing of interventions by relevant organizations to minimize social harm.

## Methods

### Model Design

A simulation was conducted using the susceptible-infected-recovered (SIR) model, which is one of the simplest and most powerful models for mathematically modeling the spread of viruses and fake news [11]. The classic formulation of the SIR model is defined by the following equations, where *S(t)* represents the number of susceptible individuals, *I(t)* represents the number of infected individuals, and *R(t)* represents the number of recovered individuals. These groups interact with each other through a disease transmission factor, contact *β*, and recover with factor *γ* within an isolated homogeneous community. The model used in this study was classified into the following 3 subgroups based on fake news belief status, assuming that the total population remains constant: S (susceptible), I (fake news believer), and R (fact-checker) [12]. The model is represented by the following equations:


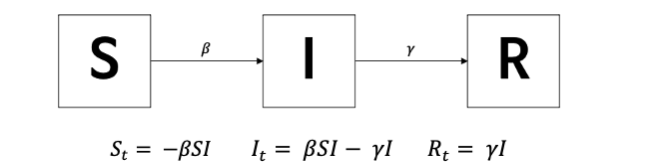


The relationships between these variables are given by the following set of equations [13]: *R_t_ +It + S_t_ = 0*.

In this study, the spread of fake news was analogized to the spread of viruses. Research on the spread of fake news using an infectious disease model has been increasing recently, as the mechanisms of spreading viruses and fake news are similar [14-17]. Specifically, individuals susceptible to fake news were classified as having the potential to trust fake news, those infected were considered fake news believers, and those recovered were identified as fact-checkers. Similar to becoming immune to disease after infection, individuals who believed in fake news, and subsequently fact-checked it, develop immunity to future misinformation.

### Parameter Setting

The parameter *β* represents the contact rate, which in this study signifies the probability of encountering fake news or the intensity of fake news diffusion. The parameter γ denotes the recovery rate, representing the probability of recovering from fake news and becoming aware of the fact. The parameters used in the model were determined based on previous studies and the situation during the fenbendazole scandal [18,19]. Typically, in the fake news propagation simulation, a contact rate (*β*) of 0.05 indicates a moderate intensity of fake news spread, where the number of individuals who believe the fake news decreases over time. This intensity is similar to the most commonly observed level in fake news spread. A low intensity (0.01) results in the fake news not spreading, whereas a high intensity (0.1) leads to a slight increase in the number of people who believe in the fake news. Thus, the fenbendazole incident can be considered as corresponding to a moderate spread due to the substantial early-stage fake news spread followed by a subsequent decline. Likewise, the recovery rate (*γ*) was set to an intermediate value of 0.05 in line with previous studies and observed propagation patterns. If the probability of fake news recovery is high (0.1), the fake news does not spread and disappears immediately at the onset. Conversely, if the probability is low (0.01), the fake news persists and does not disappear. In the case of the fenbendazole incident, the fake news spread rapidly initially but eventually subsided, indicating a moderate contact rate and recovery rate.

We utilized NetLogo 6.2.2 (Uri Wilensky) for the simulation analysis. NetLogo is a multi-agent programming language and modeling environment designed for simulating individuals and collective behaviors in complex phenomena [20]. It offers a user-friendly environment and visualization capabilities [20,21]. NetLogo serves the common goal of enabling novice programmers to develop agent-based models, which have become increasingly important and popular for studying complex systems [22].

### Ethical Considerations

Institutional review board approval was waived as the study relied on electronic data based on predetermined parameters. Additionally, the study data were deidentified due to the nature of the simulation study, thereby obviating the need for institutional review board oversight.

## Results

**Figure 3:**
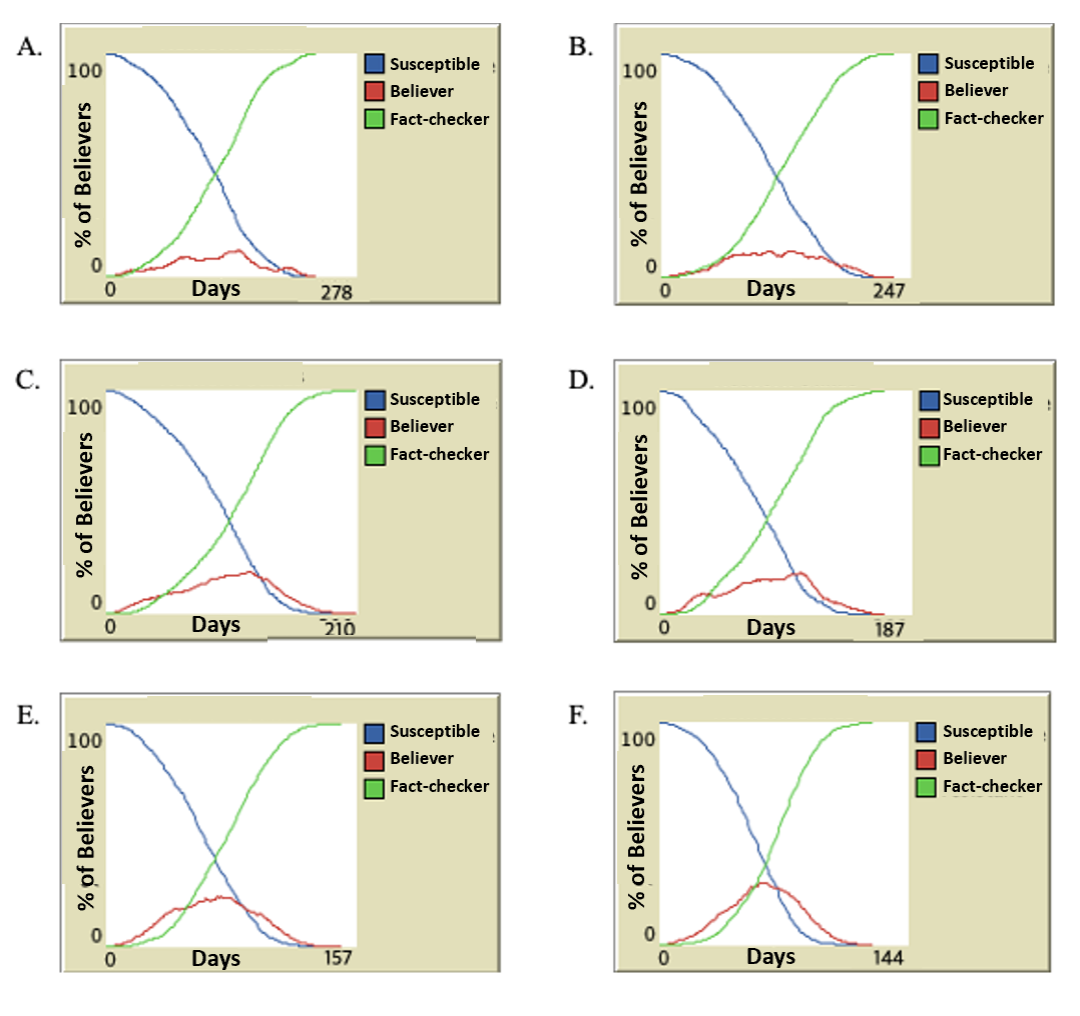
Impact of timing of intervention on fake news believers over time. The x-axis represents the days, while the y-axis indicates the percentage of fake news believers among the study population. Blue represents individuals who are susceptible to believing fake news, red indicates those who believe the fake news is true, green denotes fact-checkers who can identify the truth. (A) The results with no intervention; (B) the results after 100 days of intervention; (C) the results of an intervention on the 54th day; (D) the results of an intervention on the 16th day; (E) and (F) the results of interventions on days 10 and 5, respectively.

Figure 3 illustrates the duration of fake news spread (x-axis) and the percentage of fake news believers (the y-axis) in relation to the time of the intervention. The percentage of fake news refers to the proportion of the total population who believe in fake news. Our study focuses on patients with lung cancer, as the fenbendazole scandal primarily affected this group. Considering the 65,934 lung cancer cases in 2019, the year the fenbendazole incident occurred, we set the target population at 6000 people or approximately 10% of the total target population [23]. In the simulation study, 10% of this population was typically sampled [24].

The analysis revealed that, in the absence of interventions from relevant organizations, fake news persisted for 278 days. When interventions occurred 100 days after the scandal, the duration of the fake news spread was reduced to 247 days. An intervention on day 54, coinciding with the first report by the relevant association, reduced the duration of the fake news spread to 210 days. Further reducing the time of interventions to 16 days decreased the fake news spread duration to 187 days. When the intervention occurred on day 10, the fake news lasted for 157 days. Notably, there was no significant difference in the duration of the fake news spread when the intervention was performed within 10 days.

## Discussion

### Principal Results

This study proposes an intervention timing strategy based on the fenbendazole scandal in South Korea to prevent the spread of health-related fake news. By comparing the duration of health-related fake news under various intervention timings, the study found that intervening within at least 10 days of the occurrence of fake news significantly reduced its duration, thereby minimizing the damage caused by the spread. The study concluded that the spread of fake news could have been swiftly curtailed if relevant agencies had responded within at least 10 days following the fenbendazole scandal. However, further consideration is needed regarding how a country’s public health system can effectively identify fake news at an early stage. These findings offer significant insights into developing effective strategies to combat health-related fake news and underscore the importance of such efforts.

### Future Strategies to Stop the Spread of Fake News

The controversy surrounding the fenbendazole incident gradually diminished after interventions by the MFDS and the Korean Cancer Association. However, previous studies with a representative sample of Korean patients with cancer revealed that approximately 52% were already aware of the fenbendazole scandal, with most exposed patients having taken the drug [25,26]. This suggests that the scandal had already become a significant issue among patients with cancer, as fenbendazole can cause liver damage, highlighting the controversy’s severity [6]. The fenbendazole incident exemplifies the spread of fake information related to complementary and alternative medicine, with 85% of patients unaware of complementary and alternative medicine’s side effects and potential interactions [27]. Despite the lack of scientific evidence supporting fenbendazole’s use for cancer at first, many patients, driven by hope, self-administered it before official agencies intervened [26]. Consequently, the drug’s efficacy remains unproven, and significant side effects have been reported. Moreover, the YouTube recommendation network referenced by patients lacks reliable medical content [26,28]. Although the exact number of patients with cancer who suffered from fenbendazole intake cannot be estimated, it is evident that fake news caused irreparable damage to patients and their families.

Numerous cases of damage caused by health-related fake news, including fenbendazole, have been reported worldwide. Notably, fake news related to COVID-19 has caused significant harm. In Iran, fake news spread through SNS claimed that alcohol could cure COVID-19, leading people to consume toxic methanol. This resulted in approximately 800 deaths and 5876 injuries [29]. Similar fake news in Turkey led to 30 deaths [29]. It is estimated that at least 5800 people were hospitalized due to COVID-19 fake news between January and March 2020 [9]. Fake news can spread rapidly on SNS, and health-related misinformation can have dire consequences. Therefore, strict management of fake news is imperative.

Fake news not only confuses society but also incurs substantial social costs; therefore, it is crucial to actively prevent its spread. Recently, fake news has gained worldwide attention across various fields, with reports estimating that the annual damage caused amounts to approximately 2.74 billion dollars [30]. This issue is particularly acute with health-related fake news, which can directly influence people’s health behaviors, making the risk significant. Previous studies on fake news have underscored the need for early intervention and minimizing exposure [31]. While calculating the damage caused by fake news in direct monetary terms is challenging, the social inconvenience cost was estimated to be 620 million dollars in 2020 [32]. This is expected to rise further when considering the direct and indirect costs to society as a whole, beyond the inconvenience experienced by information consumers.

In some cases, the severity of fake news was recognized early, leading to the establishment of response systems. The fact-checking industry has grown remarkably in recent years, firmly established itself against fake news [33]. For instance, in the United States, Snopes has been providing fact-checking services since 1994, covering various fields, including politics, economics, and science [34]. Additionally, FlackCheck, launched in 2012, specifically targets fake news related to health and politics. Fact-checking involves evaluating the truthfulness of public claims and has significant potential in communications [33]. People tend to trust human fact-checking practices more in the broader context of global misinformation [35]. Therefore, it is important for each community to prevent the spread of digital misinformation through robust fact-checking.

When fake news is not properly fact-checked, people’s reliance on the media increases [36]. They gather information and form opinions through the media [37]. However, the vast amount and rapid transmission of information make it challenging to assess its reliability. Thus, developing an effective system to detect fake news is essential [38]. Governments, news media organizations, and academics worldwide are employing various strategies, including educational, legislative, and technological measures to combat fake news [39]. However, regulatory approaches to fake news have encountered strong resistance, particularly in democracies. Since 2018, the South Korean government has attempted to introduce legislation targeting fake news, but it has faced considerable opposition [40]. Nevertheless, people tend to trust the information from government and public organizations more [25]. Direct regulation of fake news may be difficult, but the government and related organizations should continue to monitor major sources of information consumption and educate the public to improve their health literacy.

### Limitations

There were some limitations of this study. First, due to the use of a simulation methodology, the results may differ from actual situations. However, simulations have been widely used across various fields to indirectly verify results in scenarios where real-life experiments are not feasible [41-44]. Furthermore, the results of this study are meaningful, as the simulations were conducted in an environment modeled on the fenbendazole incident, a representative example of cancer-related fake news, thereby reflecting the practical situation as closely as possible. Second, the effects of message factors in response to fake news, such as the message sender and message type, were not examined. Future research should include these parameters and evaluate their effect to provide richer implications for responding to fake news. Finally, this study operates under the premise that early detection of fake news is feasible. Consequently, intervening within a 10-day time frame may prove challenging if rapidly spreading false information is initially elusive to identify. It is essential for institutions to develop strategies for effective interventions. In the context of cancer-related information, given the substantial volume originating from internet cafes dedicated to cancer, it becomes imperative for relevant organizations to establish a monitoring system capable of safeguarding data and personal information while effectively surveilling such platforms.

### Conclusions

This study aimed to evaluate the timing of interventions by relevant organizations to prevent the spread of health-related fake news, using the fenbendazole case as a basis. By comparing the duration of health-related fake news under various intervention timings, the study found that intervening within at least 10 days of the initial occurrence of fake news can significantly reduce its duration, thereby minimizing the damage caused by its spread. The study concluded that if relevant agencies respond within 10 days, the spread of fake news can be swiftly suppressed. However, this study did not address how a public health system can effectively identify fake news early without infringing on freedom of expression. Combating fake news will require various strategies, including educational, legislative, and technical measures. Direct regulation of fake news remains challenging; however, considering that most information related to cancer in South Korea initially appears in cancer-related digital communities, it is essential to monitor major sources of information and educate the public to improve health information literacy.

## Abbreviations

MFDS: Ministry of Food and Drug Safety
SIR: susceptible-infected-recovered
SNS: Social Network Services

## Data Availability

The data used in the simulation can be accessed and downloaded through NetLogo. The Virus on a Network model was utilized and is available via NetLogo [45]. A detailed description of the model employed in this submission is included.
